# Transcriptome analysis reveals anthocyanin regulation in Chinese cabbage (*Brassica rapa* L.) at low temperatures

**DOI:** 10.1038/s41598-022-10106-1

**Published:** 2022-04-15

**Authors:** Yun Dai, Lei Zhang, Xiao Sun, Fei Li, Shifan Zhang, Hui Zhang, Guoliang Li, Zhiyuan Fang, Rifei Sun, Xilin Hou, Shujiang Zhang

**Affiliations:** 1 Institute of Vegetables and Flowers, Chinese Academy of Agricultural Sciences, Beijing, 100081 China; 2 State Key Laboratory of Crop Genetics and Germplasm Enhancement, College of Horticulture, Nanjing Agricultural University, Nanjing, 210095 China

**Keywords:** Physiology, Genetics

## Abstract

Chinese cabbage that prefers cold conditions is also affected by low-temperature stress, such as the accumulation of leaf anthocyanins. Research on anthocyanin biosynthesis and regulation mechanisms has made great progress. However, research on anthocyanin accumulation for resistance to biological and non-biological stress is still lacking. To study the relationship between anthocyanin accumulation of Chinese cabbage and resistance under low-temperature conditions, RNA sequencing (RNA-seq) was performed on Chinese cabbage ‘Xiao Baojian’ grown at a low temperature for four time periods and at a control temperature for five time periods. In Kyoto Encyclopedia of Genes and Genomes (KEGG) pathways, 7954 differentially expressed genes (DEGs) were enriched, of which 587 DEGs belonged to "biosynthesis of other secondary metabolites." Gene temporal expression patterns were used to discover enriched genes related to phenylpropanoid biosynthesis; flavonoid biosynthesis and anthocyanin biosynthesis pathways were found in cluster 1. The interaction networks were constructed, and hub genes were selected, showing that flavonoid biosynthesis pathway genes (*DFR*, *ANS*, *F3H*, *FLS1*, *CHS1*, *CHS3*, and *TT8*) and defense mechanisms-related genes (*DFR*, *SNL6*, and *TKPR1*) interact with each other. Anthocyanin biosynthesis DEGs in Chinese cabbage were evaluated under low-temperature conditions to map the relevant pathways, and expression maps of transcription factors in the flavonoid pathway were created at various periods. Low temperature upregulated the expression of genes related to anthocyanin biosynthesis. Taken together, our results provide further analysis of the relationship between plant anthocyanin synthesis and stress resistance and may also provide further insights for the future development of high-quality color and cold-tolerant Chinese cabbage germplasm resources.

## Introduction

Anthocyanin is a water-soluble pigment of the flavonoid family produced by secondary metabolism in plants, making plants appear red, blue, or purple. Plant pigments are not merely esthetic; their physiological functions can also play a role in disease and stress resistance^1^. Anthocyanins in fruits and vegetables can also benefit human health and reduce the risks of cancer, diabetes, and cardiovascular diseases^2^. At present, anthocyanins, as a natural pigment of various types with a wide distribution, high safety, and non-toxicity, have important applications in medicine, cosmetics, and food industries.

After decades of in-depth research, the flavonoid biosynthetic pathways in higher plants have become clear. The material needed in the early stage of flavonoid biosynthesis is phenylalanine. Phenylalanine is converted into colorless anthocyanins under the catalysis of various enzymes which are on the surface of the endoplasmic reticulum of the cytoplasm. After different degrees of hydroxylation, glycosylation, methylation, and acylation reactions, anthocyanins of various colors are formed and transported to the cell vacuoles to be enriched^3^. Two types of genes, structural and regulatory, control anthocyanin synthesis. Structural genes are used to encode a variety of enzymes required for the synthesis of anthocyanins and catalyze the intermediate and final products of the reactants to produce anthocyanins^4^. Regulatory genes are responsible for encoding special proteins, called transcription factors, which combine with regulatory structural genes to regulate the expression and intensity of structural genes^5^. Phenylalanine ammonia lyase (PAL), cinnamate 4-hydroxylase (C4H), 4-coumarate-CoA ligase (4CL), chalcone synthase (CHS), chalcone isomerase (CHI) and flavanone 3-hydroxylase (F3H), flavonoid 3'-hydroxylase (F3'H), dihydroflavonol 4-reductase (DFR), anthocyanidin synthase (ANS), and UDP-glycosyltransferase 75C1 (UGT75C1) are various enzymes in the pathway that synthesizes anthocyanins, and the enzymes encoded by these genes catalyze the synthesis of anthocyanins from the substrate^6^. These structural genes are involved in phenylpropanoid biosynthesis, flavonoid biosynthesis, and the anthocyanin biosynthetic pathway, which together promote the production of the final anthocyanins^7^. The anthocyanin synthesis pathway is mainly regulated by four transcription factor families, namely myeloblastosis (MYB), basic Helix-Loop-Helix (bHLH), WD40-repeat (WDR), and basic leucine zipper (bZIP)^8^. Studies have shown that in the *Arabidopsis* anthocyanin synthesis pathway, MYB transcription factor family genes interact with bHLH transcription factor family genes and the WD40 protein family to form a MYB-bHLH-WD40 (MBW) complex. This complex is a key regulator for the synthesis and accumulation of enriched anthocyanins^9^. In the dicot *Arabidopsis*, anthocyanin biosynthesis genes can be divided into two subgroups: early biosynthesis genes (EBGs) are activated by co-activator independent R2R3-MYB transcription factors, whereas late biosynthesis genes (LBGs) require an MBW complex^10^. EBGs of anthocyanin include *CHS*, *CHI*, *F3H*, and LBGs, such as *F3’H*, *DFR*, *ANS*, and *UGT75C1*^10,11^*.*

Previous studies have reported that plants with high anthocyanin content are resistant to biotic and abiotic stress, including salinity^12–15^, high light^16,17^, drought^18–21^, and temperature^22–28^. Salinity stress is environmental factor known to trigger anthocyanin accumulation in many plant species^14,15^, such as *Arabidopsis*^12^ and maize (*Zea mays*)^13^. Constitutive overexpression of the *Arabidopsis* glycogen synthase kinase3 (GSK3)/shaggy-like protein kinase1 (AtGSK1) in transgenic plants was shown to induce salt stress responses, including the accumulation of anthocyanins^12^. High light intensity stimulates the production of anthocyanins in various plant species, and tomatoes under high light conditions showed more intense anthocyanin pigmentation than tomatoes grown in the shade^29^. High light mainly regulates anthocyanin production by controlling R2R3-MYB transcription factors, and blue and red light can induce the high expression of *CHS* in petunia flowers^17^. Many studies have shown that drought induces anthocyanin synthesis in plants. For example, drought conditions trigger the transcription of a series of anthocyanin biosynthesis genes in *Arabidopsis*, potato (*Solanum tuberosum* L.), and wheat (*Triticum aestivum* L.), which directly promotes the accumulation of anthocyanins^18–20^. Ethylene response factor 38 (ERF38) interacts with MYB1, a positive regulator of anthocyanin synthesis, and responds to drought stress^21^. The effect of temperature on many plants was two-sided; low temperature promotes the accumulation of anthocyanins in plants, and high temperature reduces their accumulation. In *Arabidopsis*, anthocyanins were induced by low temperatures^22^ and reduced by high temperatures^23^. In apple (*Malus* × *domestica* Borkh.) and pear (*Pyrus communis* L.), low temperatures increased both anthocyanin content and the expression of genes of the anthocyanin biosynthetic pathway^24–26^. The expression of anthocyanin biosynthetic genes *C4H, F3H, DFR, ANS,* and *UFGT* was enhanced under low temperature treatment^27^. Under low temperatures, *MdMYBA* binds specifically to ANS and activates anthocyanin accumulation in apple skin^28^. Research on the biosynthesis and regulation mechanism of anthocyanins has also made great progress, and the influence of various factors on the synthesis of anthocyanins has gradually been studied and reported. However, research on anthocyanin accumulation for resistance to biological and non-biological adversities is still lacking.

Chinese cabbage (*Brassica rapa* L. ssp. *pekinensis*), a leafy vegetable belonging to the cruciferous family, is also known as heading cabbage or wrapping cabbage. It has a long history of cultivation and is a specialty vegetable in China. The leaf color of Chinese cabbage is basically green. The leaf color of the varieties grown in the north is mostly dark green, and the leaf color in the south is light green. In recent years, the cultivation of purple Chinese cabbage has increased gradually. Purple cabbage is selected by crossbreeding purple cabbage and high-quality Chinese cabbage through traditional breeding techniques. When green Chinese cabbage was exposed to low temperatures, the leaves of the green Chinese cabbage gradually turned purple. To study anthocyanin regulatory pathways and related genes in Chinese cabbage under low-temperature conditions, the highly inbred line material ‘Xiao Baojian’ (XBJ) was adopted to conduct RNA-seq in four low-temperature periods and five periods at the control temperature. Temporal expression patterns showed that flavonoid biosynthesis pathway genes (*DFR*, *ANS*, *F3H*, *FLS1*, *CHS1*, *CHS3*, and *Transparent testa 8* (*TT8)*) and defense mechanism-related genes (*DFR*, *cinnamoyl-CoA reductase-like SNL6* (*SNL6)*, and *tetraketide alpha-pyrone reductase 1* (*TKPR1)*) interact with each other, providing new insights into the relationship between plant anthocyanin synthesis and stress resistance mechanisms.

## Materials and methods

### Plant materials and treatments

Highly inbred line material, ‘Xiao Baojian’ (XBJ), was provided by the Institute of Vegetables and Flowers of the Chinese Academy of Agricultural Sciences located in Beijing, China (39°56’ N, 116°20’ E), and used for RNA sequencing (RNA-seq) analysis. Plants were initially grown for 32 days in the nursery greenhouse (25 ± 2 °C under natural light) of the Institute of Vegetables and Flowers of the Chinese Academy of Agricultural Sciences. Subsequently, plants were treated under normal conditions (25 °C under a 16/8 h light/dark photoperiod and 150 µmol m^−2^ s^−1^ light intensity) for 0, 15, 25, 35, or 45 days (N-0DAT, N-15DAT, N-25DAT, N-35DAT, N-45DAT; days after normal treatment) or low-temperature conditions (4 °C under a 16/8 h light/dark photoperiod and 150 µmol m^−2^ s^−1^ light intensity) for 15, 25, 35, or 45 days (L-15DAT, L-25DAT, L-35DAT, L-45DAT; days after low-temperature treatment). The plant studies were carried out in accordance with relevant institutional, national, and international guidelines and legislation. Leaves of plants in each treatment period were collected, frozen in liquid nitrogen, and stored at −80 °C for RNA extraction.

### Determination of total anthocyanin content

Total anthocyanin content was measured via the micromethod using the Plant Anthocyanin Content Detection Kit (Beijing Solarbio Science & Technology Co., Ltd., Beijing, China). The absorbance of the samples were determined at 500 nm. A standard curve was obtained according to the kit instructions, with the equation y = 0.1108x + 0.0171 (R^2^ = 0.9935). A sample mass of 0.1 g was used to calculate ∆A = A measuring tube—A blank tube. ∆A was used as y in the standard curve to obtain x. The total anthocyanin content was calculated as content (mg/g) = x/0.1. All operations were conducted with three repeats.

### RNA extraction and RNA sequencing (RNA-seq)

To reveal the molecular mechanisms of anthocyanin metabolism at low-temperature conditions, 27 cDNA sequencing libraries (9 treatments × 3 replicates) were constructed and sequenced using NEBNext® Ultra™ RNA Library Prep Kit for Illumina (New England Biolabs, Ipswich, MA, USA) following the manufacturer’s recommendations. mRNA was purified from total RNA using magnetic beads with poly-T oligomers, and the library fragments were purified using the AMPure XP system (Beckman Coulter, Brea, CA, USA). In-house perl scripts were used to remove reads containing adapters, reads containing ploy-N, and low-quality reads from the raw data to obtain clean data (clean reads). RNA-seq analysis of 27 samples generated 1,183,051,830 total reads, 591,525,915 clean reads, and 176.89 Gb clean data. The Q30 was ≥ 91.50%, and the average GC content was 46.22%. The clean reads of each sample were compared with the designated *B*. *rapa* reference genome (v 3.0)^30^, and the comparison efficiency ranged from 70.57 to 90.87% (Supplementary Table S1). This indicates the credibility of the RNA-seq data.

### Differentially expressed gene analysis and unigene annotation

The expression level of unigenes was estimated using Cufflinks v2.2.1^31^, and the relative abundance in each sample was normalized to fragments per kilobase per million reads (FPKM). DESeq2^32^ was used to perform differential expression analysis of two samples. Benjamini and Hochberg’s approach^33^ was used to control the false discovery rate to adjust the obtained *P*-value. Genes with adjusted *P*-values < 0.01 found by DESeq2 were designated as differentially expressed.

Unigene functions were annotated based on the gene ontology (GO) enrichment analysis using GOseq R software^34^. Kyoto Encyclopedia of Genes and Genomes (KEGG)^35^ was used to test the statistical enrichment of genes. NCBI (National Center for Biotechnology Information) non-redundant protein sequences (Nr), protein family (Pfam), Clusters of Orthologous Groups of proteins (KOG/COG), Swiss-Prot (a manually annotated and reviewed protein sequence database), and eggNOG (Non-supervised Orthologous Groups) were used to annotate the genes.

### Gene temporal expression and hub gene analysis

To evaluate the gene expression patterns of Chinese cabbage in low-temperature conditions, expression pattern analysis was performed. In total, 22,095 differentially expressed genes (DEGs) in the comparisons of 0DAT vs. L-15DAT, N-15DAT vs. L-15DAT, N-25DAT vs. L-25DAT, N-35DAT vs. L-35DAT, and N-45DAT vs. L-45DAT were used to cluster the change patterns of unigenes through the short time-series expression miner (STEM) software^36^, STEM uses gene expression for clustering, expression and visualization, and clusters genes with similar expression trends together into different clusters. The 22,095 DEGs were clustered into 14 expression profiles. The clustered profile of DEGs with *p*-value < 0.01 is considered to be significantly different from the reference set for each sample.

Hub genes are good representatives of each cluster and have important biological significance in system analysis. Hub genes are genes with the most connection points in each module and can be used to predict the importance and function of genes. The sequences of the clustered DEGs were blasted to the *B*. *rapa* reference genome (v 3.0) to obtain the predicted interaction networks of these DEGs. The protein–protein interactions were identified in the STRING database (http://string-db.org/). Then, the interaction networks of these DEGs were visualized in Cytoscape, and the top 20 hub genes were calculated and ranked based on the MCC method using the novel Cytoscape plugin cytoHubba^37^.

### Quantitative real-time PCR (qRT-PCR) analysis

Fourteen genes involved in the regulatory pathway of anthocyanin synthesis were randomly selected for qRT-PCR analysis, and two known genes were also selected to express trends for qRT-PCR analysis. Primers were designed with Primer v5.0. *Actin* was used as an internal control for gene expression (Supplementary Table S2). The QuantStudio 12 K Flex qPCR System (ThermoFisher Scientific, Waltham, MA, USA) was applied for qRT-PCR, and ChamQ Universal SYBR qPCR Master Mix (Vazyme Biotech Co., Ltd., Nanjing, China) was used for qRT-PCR reactions. The 2^−ΔΔCt^ method was used to calculate gene expression levels^38^.

### Statistical analyses

The data were analyzed with SPSS v 19.0 (SPSS, Chicago, IL, USA), using a one-way analysis of variance (ANOVA) with Duncan's multiple range post hoc test and a significance threshold of *p* < 0.05. The results were graphed using Sigmaplot v 10.0 (Systat Software Inc., San Jose, CA, USA) for visualization.

## Results

### Appearance of anthocyanins and anthocyanin content of Chinese cabbage at low temperatures

**Figure 1:**
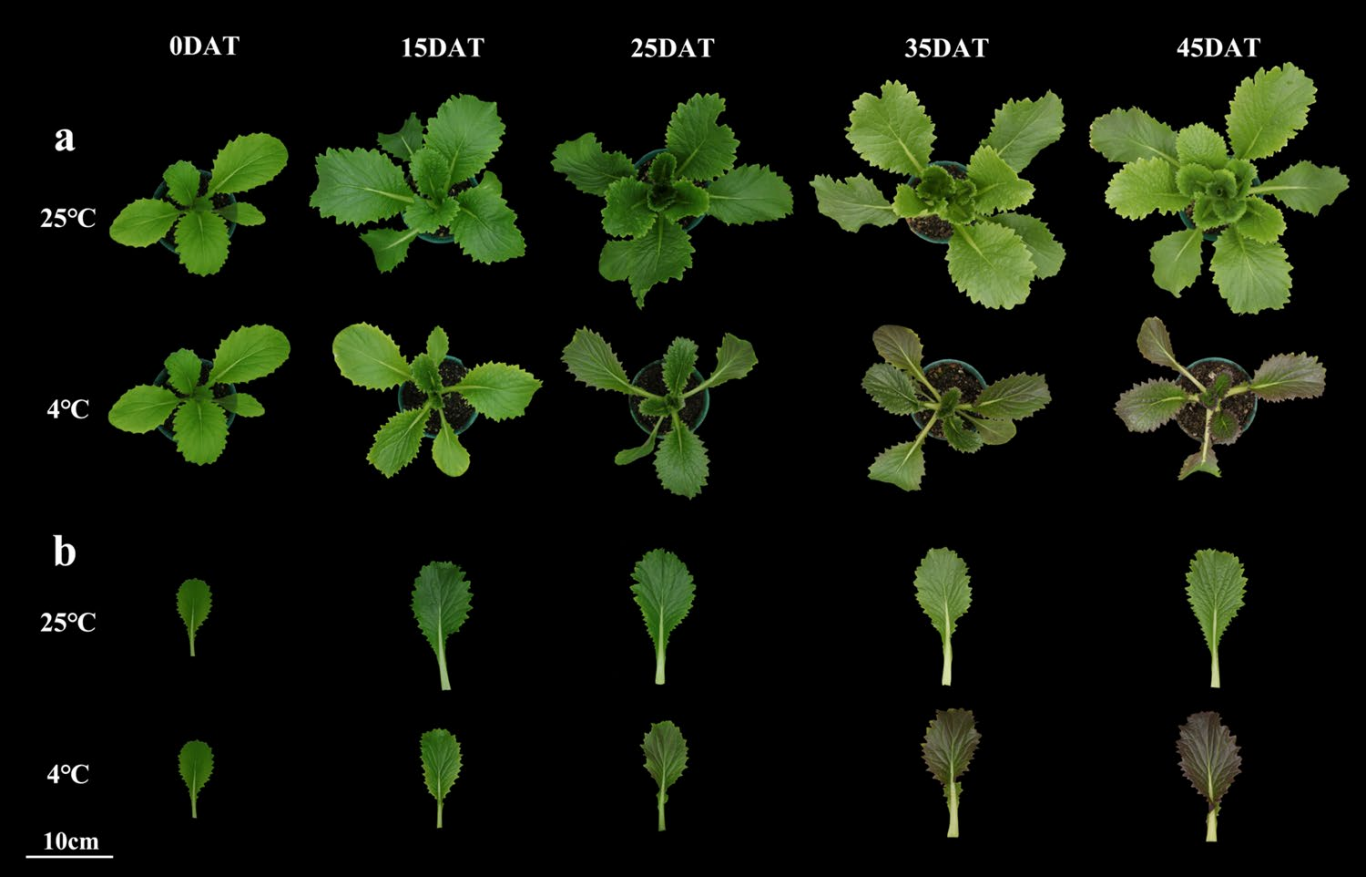
Typical phenotypes under 25 °C and 4 °C at 0, 15, 25, 35, and 45 days after treatment (DAT). (**a**) top view, (**b**) leaf view.

**Figure 2:**
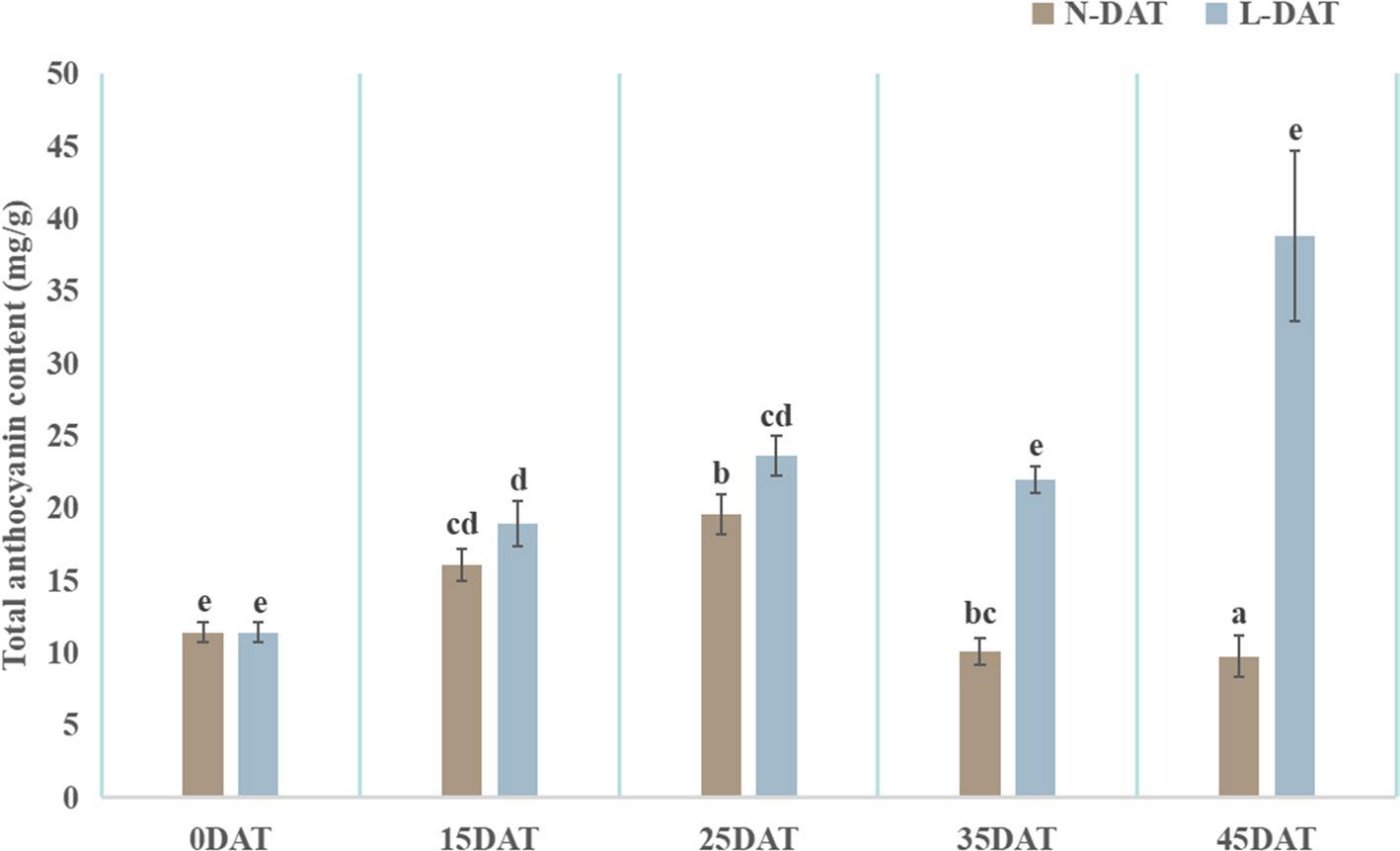
Total anthocyanin content in ‘Xiao Baojian’ (XBJ) in different treatments. Error bars represent the SE (*n* = 3). Values with the same letter are not significantly different at *p* < 0.05. N-DAT represents days after normal treatment, and L-DAT represents days after low-temperature treatment.

To observe the phenotypic changes in Chinese cabbage under low-temperature conditions, plants were observed (Fig. 1) in each low-temperature treatment and the control. Compared with the control, plants gradually showed symptoms of cold stress after low-temperature treatment. Leaf growth was stunted, and plant’s growth trend was inhibited under low-temperature conditions. The plants were shorter, and the leaves gradually turned purple as the low-temperature conditions persisted. To analyze the anthocyanin components, the total anthocyanin content was measured in the low-temperature treatments and in the controls. The anthocyanin content of Chinese cabbage under low-temperature conditions was greater than that in the control. As low temperatures persisted, the anthocyanin content first increased (15 and 25 DAT), and then slowly decreased (35 DAT) before increasing sharply (45 DAT) (Fig. 2). At 35 and 45 DAT, the anthocyanin content under low-temperature conditions began to be significantly greater than that under normal conditions (Fig. 2), which was consistent with the phenotypes of 35 and 45 DAT (Fig. 1). At 25 DAT, Chinese cabbage under low-temperature conditions began to have a purple leaf phenotype. In addition, the degree of sensory purple at 45 DAT was greater than at 35 DAT, indicating that persistently low temperatures increased anthocyanin accumulation, causing increasingly purple leaves. Changes in the molecular mechanism may occur at the onset of low temperatures and later affect the phenotype.

### RNA-seq analysis

**Figure 3:**
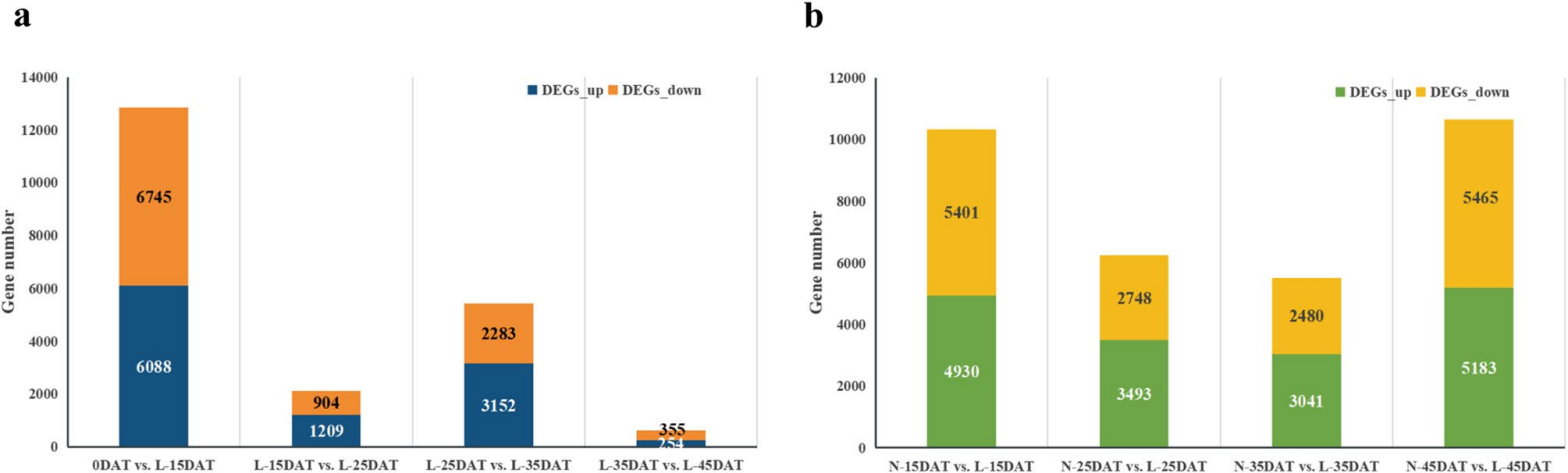
The number of differentially expressed genes (DEGs) between eight comparable groups (0DAT vs. L-15DAT, L-15DAT vs L-25DAT, L-25DAT vs L-35DAT, L-35DAT vs L-45DAT, N-15DAT vs L-15DAT, N-25DAT vs L-25DAT, N-35DAT vs L-35DAT, N-45DAT vs L-45DAT). *p* < 0.01 and |log_2_ FC|> 1.5 were used as cut-of criteria for significance.

To identify the DEGs between each treatment and the control, DEG analysis was implemented. The transcription level of the gene was presented as the FPKM value. Two groups, each with four comparisons, were analyzed, namely, different low temperature comparisons of 0DAT vs. L-15DAT, L-15DAT vs. L-25DAT, L-25DAT vs. L-35DAT, and L-35DAT vs. L-45DAT (Fig. 3a) and the same period comparisons of N-15DAT vs. L-15DAT, N-25DAT vs. L-25DAT, N-35DAT vs. L-35DAT, and N-45DAT vs. L-45DAT (Fig. 3b). All time points showed similar numbers of upregulated and downregulated genes. In different low temperature comparisons, 6745 upregulated and 6088 downregulated genes were identified in 0DAT vs. L-15DAT, indicating that most of the cold-regulated genes were early response genes. In comparisons between the same time period, N-15DAT vs. L-15DAT and N-45DAT vs. L-45DAT showed more upregulated and downregulated genes than N-25DAT vs. L-25DAT and N-35DAT vs. L-35DAT, indicating that many cold-regulated genes were also gradually regulated.

**Figure 4:**
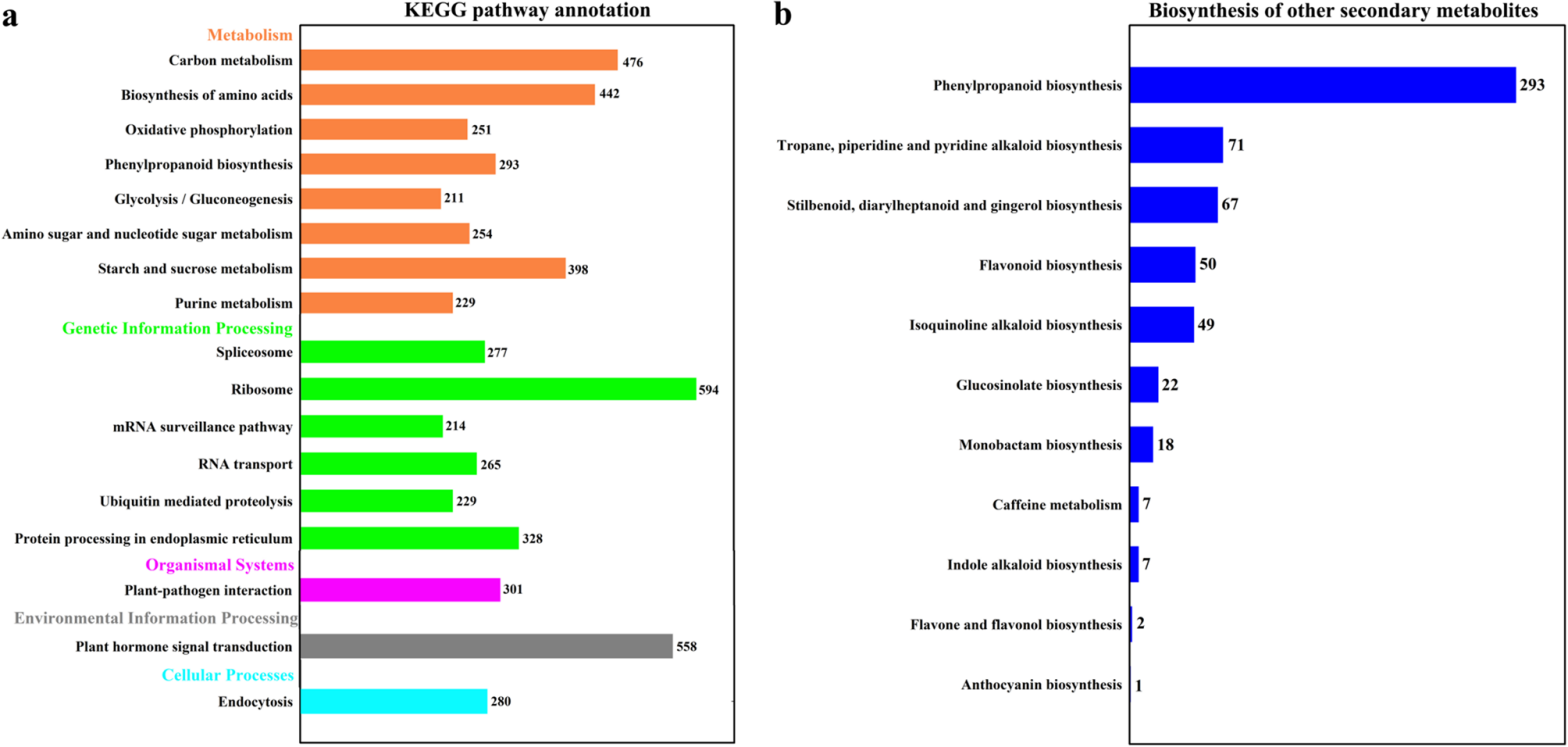
KEGG pathway classification of DEGs. (**a**) Selected KEGG pathways with more than 200 genes for mapping. (**b**) Biosynthesis of other secondary metabolites related genes KEGG distribution.

After removing redundant genes, 32,974 DEGs were obtained, which were then mapped to KEGG pathways. Of these DEGs, 7954 (24.12%) were mapped, and of those with KEGG pathways over 200 genes were selected for mapping, forming 5 categories and 17 subcategories (Fig. 4a). The most abundant category was "metabolism," followed by "genetic information processing." In "metabolism," the most abundant subcategories were "carbon metabolism," "biosynthesis of amino acids," and "starch and sucrose metabolism," with enrichments of 476, 442, and 398 DEGs, respectively. The richest subcategory in "genetic information processing" was "ribosome" with 594 DEGs. "Plant hormone signal transduction" was also an enriched subcategory with 558 DEGs. Notably, there were 587 DEGs belonging to "biosynthesis of other secondary metabolites," among which the "phenylpropanoid biosynthesis" (ko00940) contained 293 DEGs, "flavonoid biosynthesis" (ko00941) contained 50 DEGs, and "anthocyanin biosynthesis" (ko00942) contained 1 DEG (Fig. 4b).

### Gene temporal expression patterns and hub gene selection

**Figure 5:**
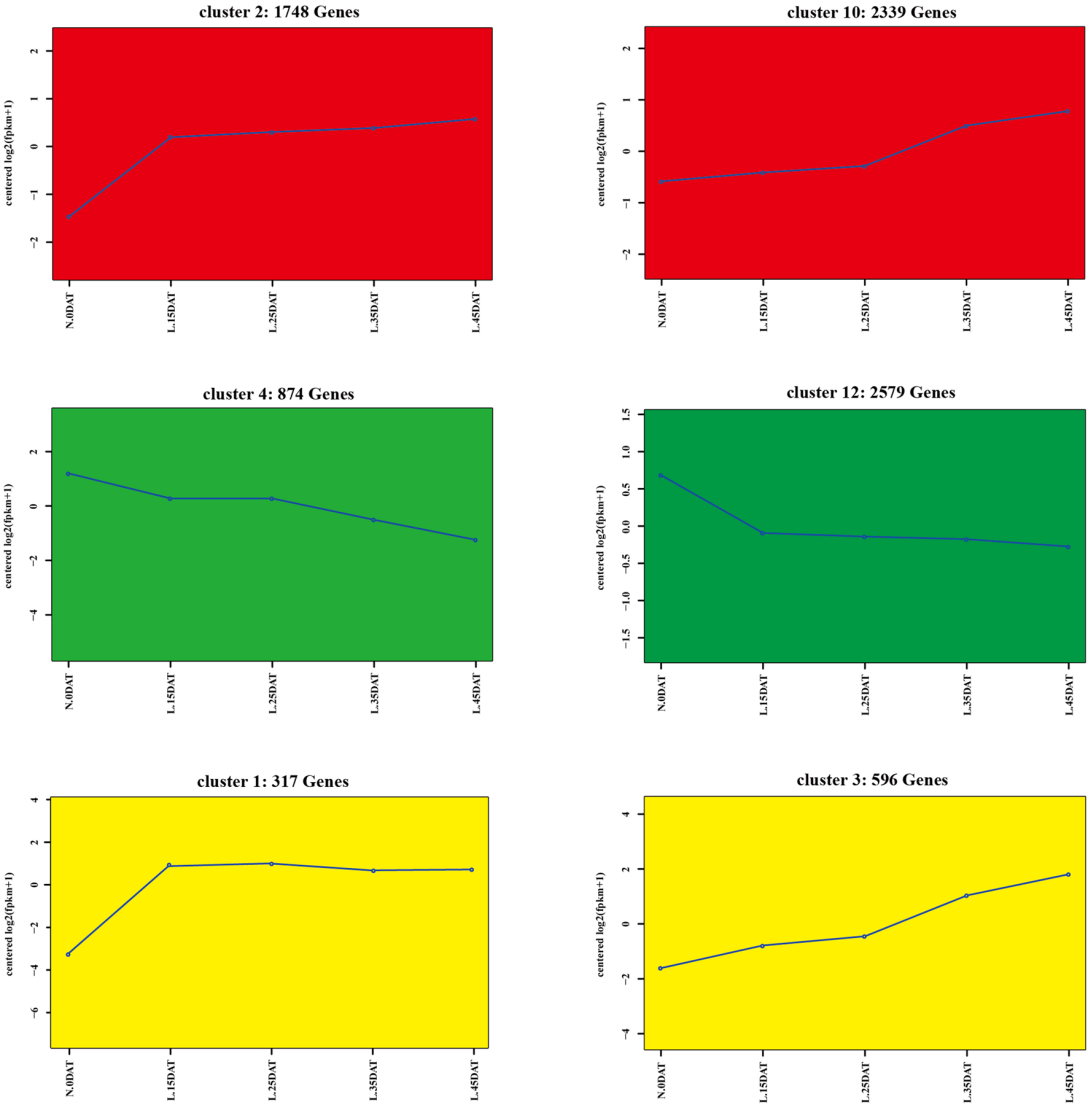
Important expression clusters of DEGs in low-temperature conditions. Each square represents one cluster. Clusters 2 (1748 genes) and 10 (2339 genes) exhibited a state of continuous induction during low temperature persistence; clusters 4 (874 genes) and 12 (2579 genes) exhibited a state of continuous decrease during low temperature persistence; cluster 1 (317 genes) exhibited enriched phenylpropanoid (ko00940), flavonoid (ko00941), and anthocyanin biosynthesis (ko00942) pathways-related genes; and cluster 3 (596 genes) enriched flavonoid biosynthesis (ko00941) pathway-related genes. The pathways were constructed based on the KEGG pathway (ko00940, ko00941 and ko00942)^35^ and previous works. The x-axis represents treatment time, and the y-axis represents expression.

**Figure 6:**
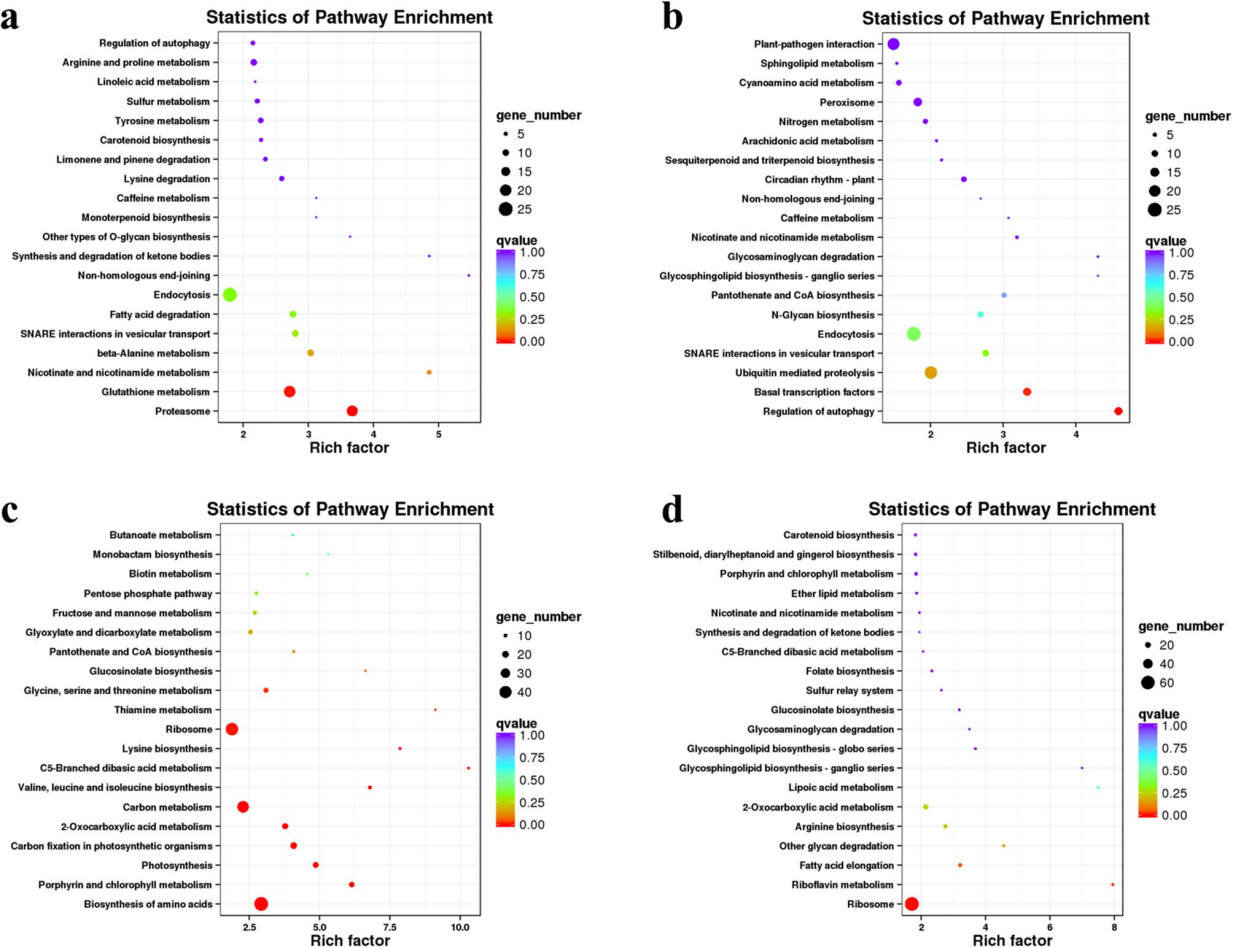
KEGG pathway enrichment of clusters (**a**) 2, (**b**) 10, (**c**) 4, and (**d**) 12. The size of the circle represents the number of genes enriched in the pathway, and the depth of the color represents the size of the *q* value.

To determine the main transcriptional dynamics related to continuous low temperature in Chinese cabbage, cluster analysis of DEGs was conducted using STEM software. In total, 22,095 DEGs were classified into 14 clusters with similar expression profiles (Fig. 5 and S1). In clusters 2 and 10, 1748 and 2339 genes, respectively, exhibited a state of continuous induction during low temperature persistence. DEGs that continued to decrease with persistent low-temperature conditions were enriched in clusters 4 (874 genes) and 12 (2579 genes). KEGG enrichment analysis was performed on these four clusters to fully understand their biological relevance. Many genes in clusters 2 and 10 were classified together into "regulation of autophagy," "SNARE interactions in vesicular transport," "endocytosis," "nicotinate and nicotinamide metabolism," "caffeine metabolism," and "non-homologous end-joining" (Fig. 6a, b; Tables S3 and S4). This indicated that continuous low temperatures promote the continuously high expression of genes in these pathways to deal with the harm caused by low temperatures. "Porphyrin and chlorophyll metabolism," "2-oxocarboxylic acid metabolism," "C5-branched dibasic acid metabolism," "ribosome," and "glucosinolate biosynthesis" were jointly enriched in clusters 4 and 12, indicating that genes enriched in these pathways had continuously low expression at low temperatures (Fig. 6c, d; Supplementary Tables S5 and S6).

**Figure 7:**
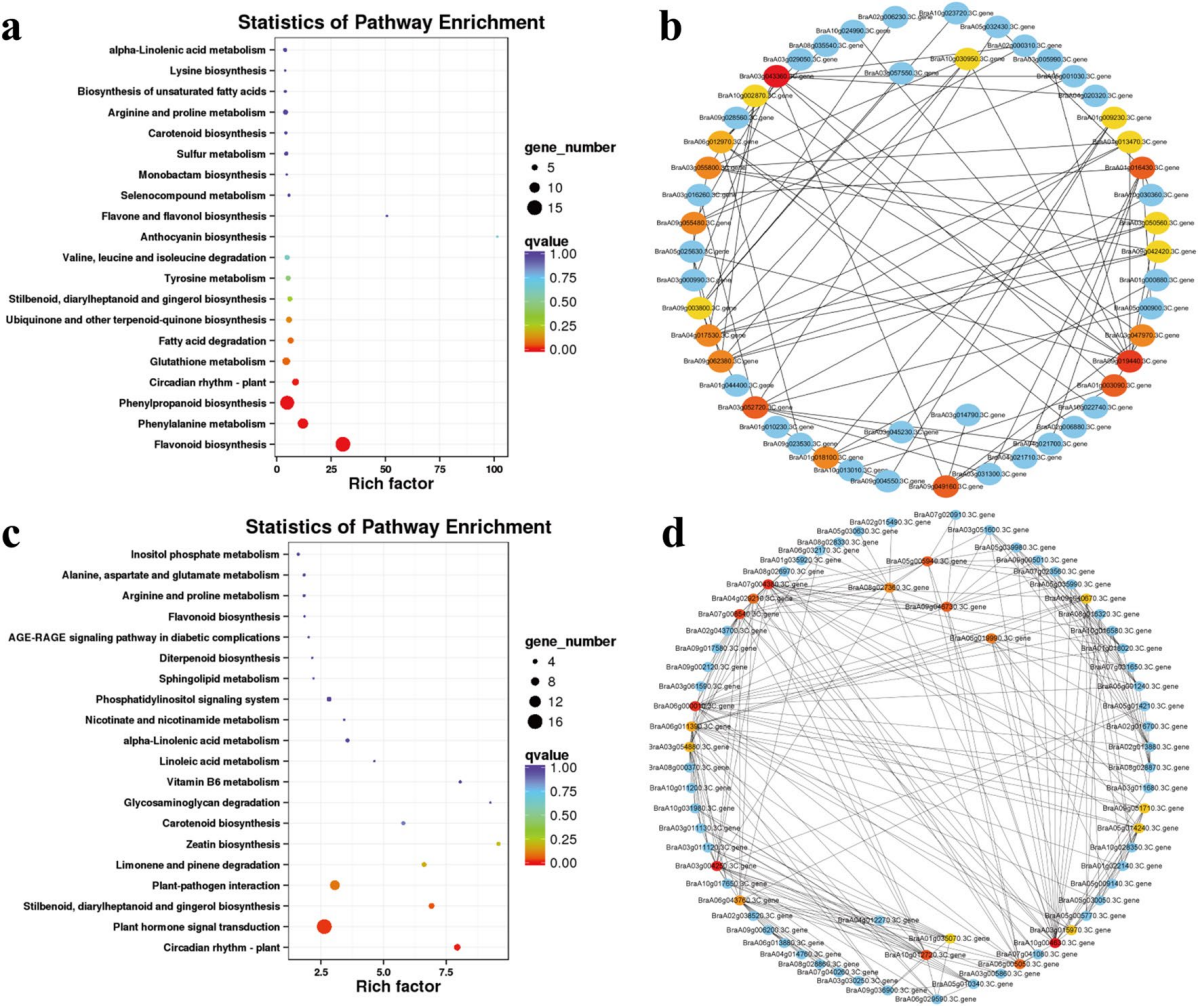
KEGG pathway enrichment and putative interaction networks of clusters 1 and 3. (**a**) KEGG pathway enrichment of cluster 1, (**b**) putative interaction networks of cluster 1, (**c**) KEGG pathway enrichment of cluster 3, and (**d**) putative interaction networks of cluster 3. From red to orange to yellow represent the top 20 hub genes with a correlation from strong to weak, and blue represents other genes associated with hub genes. The size of the circle represents the number of genes enriched in the pathway, and the depth of the color represents the *q* value (**a** and **c**).

Notably, phenylpropanoid (ko00940), flavonoid (ko00941), and anthocyanin biosynthesis (ko00942) pathways were found in cluster 1 (Fig. 5) of the KEGG annotation pathway (Supplementary Table S7). These three pathways were the processes that must be experienced for anthocyanins to be synthesized (Fig. 7a). The flavonoid biosynthesis pathway (ko00941) was enriched in cluster 3 (Figs. 5; 7c; Supplementary Table S8). The interaction networks of DEGs were screened in clusters 1 and 3, and the top 20 genes that were representative of the clusters were selected, thereby providing the most detailed information for further research (Fig. 7b, d).

**Figure 8:**
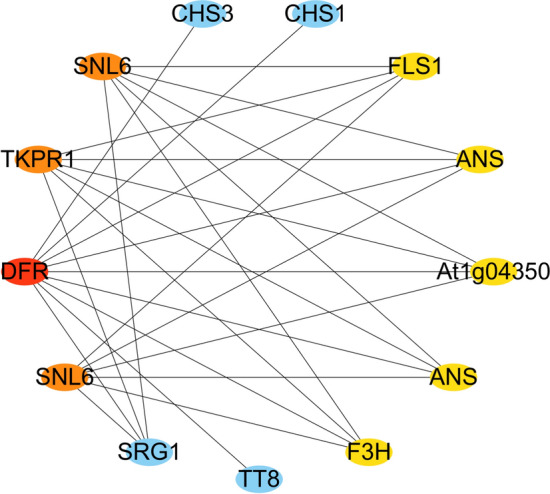
The putative interaction networks of genes associated with hub genes related to flavonoid biosynthesis (ko00941) in cluster 1. From red to orange to yellow represent the top 20 hub genes with a correlation from strong to weak, and blue represents other genes associated with hub genes.

KEGG pathway annotation in cluster 1 showed five hub genes related to flavonoid biosynthesis (ko00941): BraA09g042420.3C.gene (*F3H*), BraA09g019440.3C.gene (*DFR*), BraA01g013470.3C.gene (*ANS*), BraA10g030950.3C.gene (*FLS1*, flavonol synthase 1), and BraA03g050560.3C.gene (*ANS*). Among them, *DFR*, *ANS*, and *F3H* were essential regulatory genes in the anthocyanin synthesis pathway (ko00942) (Supplementary Table S9). Flavonoid biosynthesis pathway genes (*DFR*, *ANS*, *F3H*, *FLS1*, *CHS1*, and *CHS3*) interacted with transcription factor TT8 of the anthocyanin synthesis pathway (Fig. 8). Moreover, the *At1g04350* gene was annotated in the "secondary metabolites biosynthesis pathway" and "defense mechanism" (*DFR*, *SNL6*, and *TKPR1*) of the KOG annotation (Table 1). The above results indicated that "secondary metabolites biosynthesis pathway" and "defense mechanisms" were closely related under cold stress. KOG annotation in cluster 3 revealed that hub genes were enriched in "posttranslational modification, protein turnover, chaperones" and "signal transduction mechanisms," and no hub genes related to the anthocyanin synthesis pathway were found (Supplementary Table S10).

**Table 1:** Hub gene annotation of cluster 1. KEGG: Kyoto Encyclopedia of Genes and Genomes; KOG/COG: Clusters of Orthologous Groups of proteins.

ID	Gene name	KEGG_pathway	KOG/COG_class
BraA09g019440.3C.gene	*DFR*	Flavonoid biosynthesis	Defense mechanisms
BraA01g013470.3C.gene	*ANS*	Flavonoid biosynthesis	Secondary metabolites biosynthesis
BraA03g050560.3C.gene	*ANS*	Flavonoid biosynthesis	Secondary metabolites biosynthesis
BraA09g042420.3C.gene	*F3H*	Flavonoid biosynthesis	Secondary metabolites biosynthesis
BraA04g017530.3C.gene	*SNL6*	–	Defense mechanisms
BraA03g055800.3C.gene	*SNL6*	–	Defense mechanisms
BraA09g062380.3C.gene	*TKPR1*	–	Defense mechanisms
BraA10g030950.3C.gene	*FLS1*	Flavonoid biosynthesis	Secondary metabolites biosynthesis
BraA10g002870.3C.gene	*At1g04350*	–	Secondary metabolites biosynthesis
BraA09g028560.3C.gene	*TT8*	–	–
BraA05g025630.3C.gene	*SRG1*	–	Secondary metabolites biosynthesis
BraA10g024990.3C.gene	*CHS1*	Flavonoid biosynthesis	–
BraA03g005990.3C.gene	*CHS3*	Flavonoid biosynthesis	–

### Modulation of anthocyanin synthesis pathway genes under low-temperature conditions in Chinese cabbage

**Figure 9:**
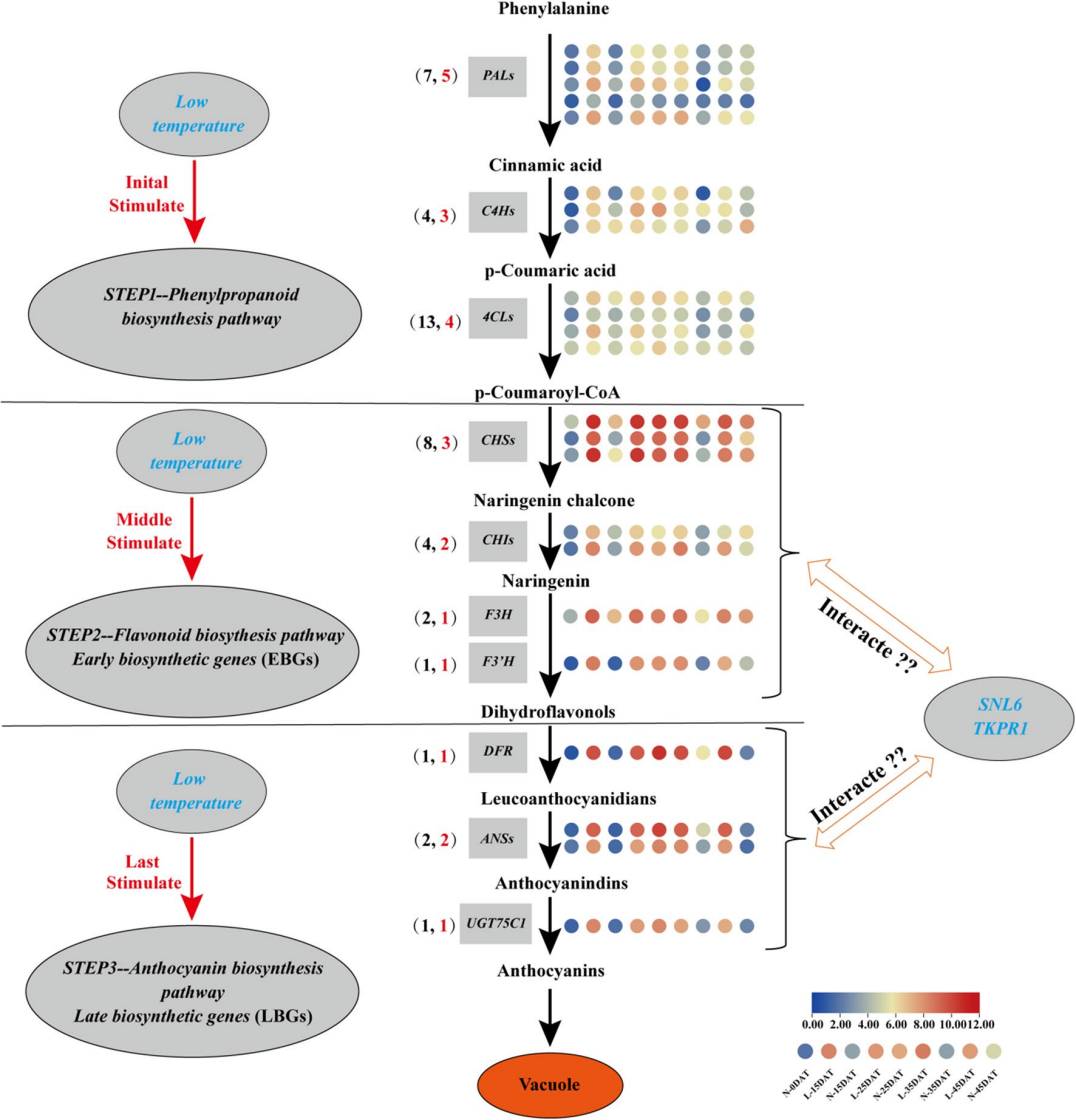
Potentially related anthocyanin biosynthesis pathways under low-temperature conditions in Chinese cabbage. The heat map was generated using TBtools software, and the nine circles in each horizontal row correspond to nine treatments. The gray boxes indicate different genes. The numbers in parentheses indicate the number of genes, with the total number in black font and the number of DEGs in red font.

The anthocyanin synthesis pathway was the main branch of the phenylpropanoid pathway. From phenylalanine to anthocyanin, there were three main steps in the biosynthesis pathway: phenylpropanoid biosynthesis (ko00940), flavonoid biosynthesis (ko00941), and the anthocyanin biosynthesis (ko00942) pathway. *PAL*, *C4H*, and *4CL* are phenylpropanoid pathway genes and are also the starting point for the synthesis of flavonoids. In this study, the expression of 7 *PALs*, 5 of which were DEGs, under low-temperature conditions was higher than the control expression levels at the same period (Fig. 9; Supplementary Table S11). Three of the four *C4Hs* were DEGs, and two of them were more highly expressed in the low-temperature period than in the control period. Similarly, 13 *4CLs* were identified, but only 4 were DEGs, which had higher expression levels during the low-temperature period. This suggested that the phenylpropanoid pathway upstream of anthocyanin biosynthesis was more active during low-temperature conditions. Early anthocyanin biosynthesis genes (EBGs) were flavonoid biosynthesis genes, including *CHS*, *CHI*, and *F3H*. Three of the eight *CHS* genes were identified as DEGs, and two of four *CHIs* were identified as DEGs; one of two *F3H* genes were DEGs. These DEGs exhibited higher expression levels in L-15DAT and L-35DAT. *F3’H*, *DFR*, *ANS*, and *UGT75C1* are LBGs. In this study, 1 *F3'H*, 1 *DFR*, 2 *ANS*, and 1 *UGT75C1* were upregulated DEGs, and the expression level in low-temperature conditions was much higher than the control except at 25 DAT. Low temperature promoted the increase in the expression of related anthocyanin synthesis genes. This phenomenon suggests that anthocyanin synthesis may play a pivotal role in the regulation of molecular responses in plants under cold stress conditions.

### MYB transcription factor identification

**Figure 10:**
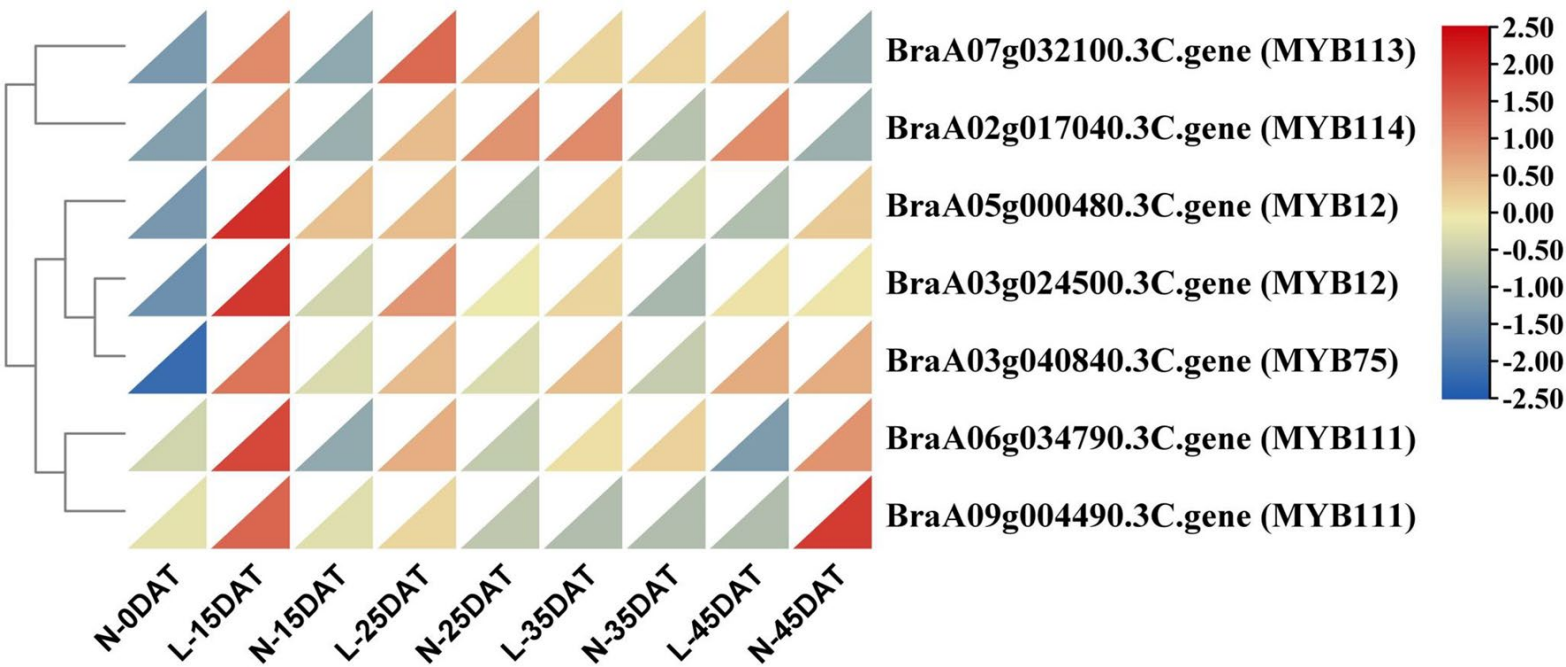
Heat map generated based on the FPKM of seven putative MYBs in nine different treatments. The x-axis represents treatment time, and the y-axis represents gene ID.

Gene transcription of the flavonoid pathway is regulated by transcription factors, including the MYB gene family, and plays a central role in the regulation of anthocyanin biosynthesis. In the present study, seven differentially expressed MYB transcription factors were obtained (Fig. 10; Supplementary Table S12). These MYB genes, as DEGs, have a higher expression level in low-temperature conditions than in normal conditions, especially at L-15DAT vs. N-15DAT and L-35DAT vs. N-35DAT. It is speculated that the expression of the L-15DAT MYB genes were the highest due to the first stimulation of low temperature, and that they would decrease over time. Taken together, these MYBs require in-depth study of the anthocyanin synthesis pathway in Chinese cabbage under low-temperature conditions.

### qRT-PCR validation of selected related genes

**Figure 11:**
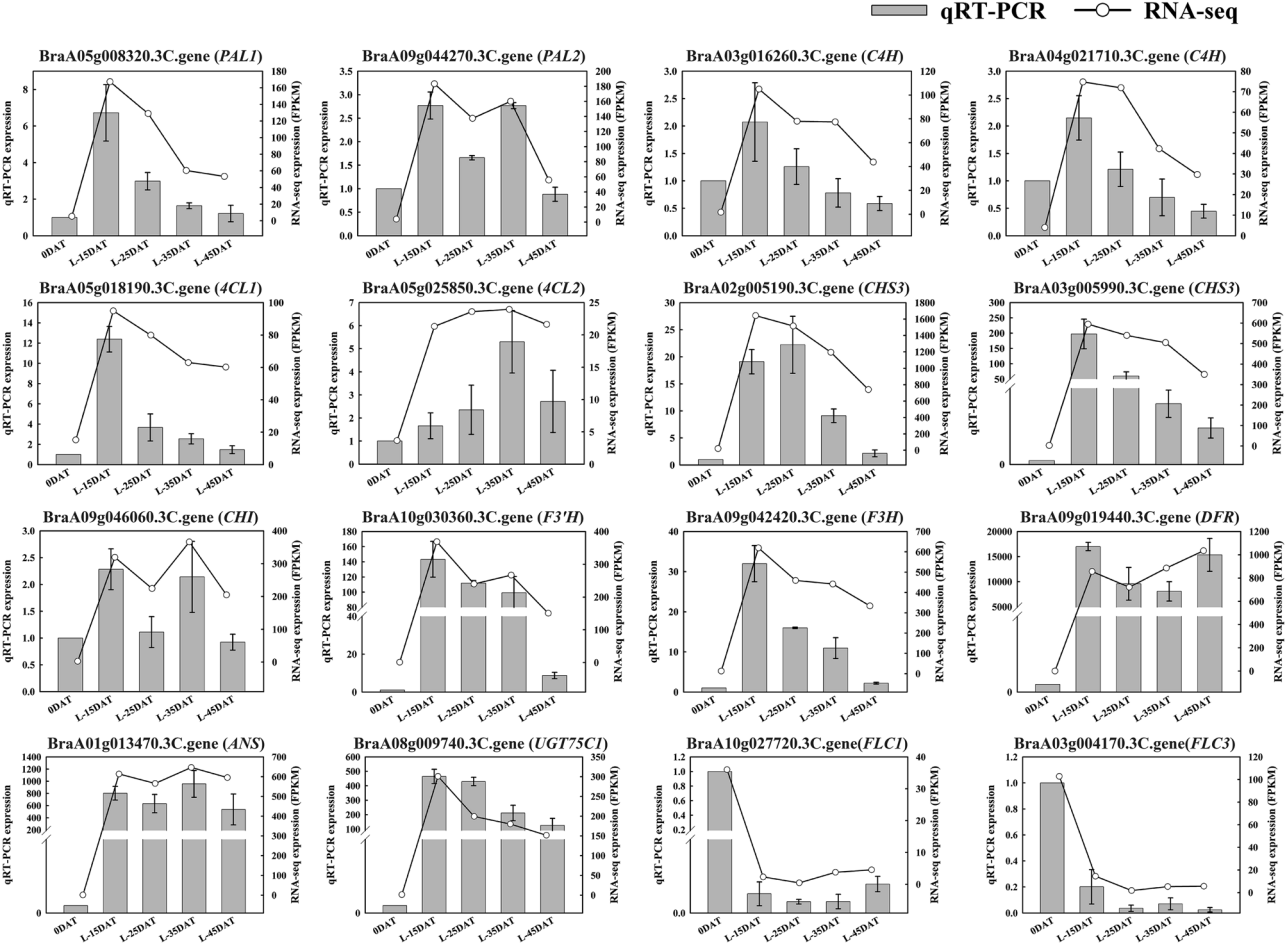
qRT-PCR was performed using 14 DEGs of the anthocyanin synthesis pathway. Bar and line graphs represent the qRT-PCR and RNA-seq data, respectively. Data are presented as the mean ± standard error (SE).

The transcription levels of 14 DEGs related to the anthocyanin synthesis pathway were evaluated using qRT-PCR. RNA-seq and qRT-PCR results were consistent during the low temperature process (Fig. 11), indicating the reliability of high-throughput transcriptome sequencing. The accuracy of the qRT-PCR works was again validated for two genes (BraA10g027720.3C.gene *FLC1*; BraA03g004170.3C.gene *FLC3*) with an overall downward trend at low temperatures^39^. During the low-temperature period, the expression levels of anthocyanin synthesis-related genes were higher than those in the control, demonstrating that low temperatures affect the anthocyanin synthesis pathway in Chinese cabbage.

## Discussion

Many previous studies have indicated that plant anthocyanins are not always synthesized in normal and suitable growth conditions. Generally, anthocyanins are induced under abnormal stress, such as high light, nutrient consumption, and cold stress^40–42^. In fruits and vegetables, such as grape (*Vitis vinifera* L.)^43^, blood orange (*Citrus sinensis* L. Osbeck)^44^, apple (*Malus* × *domestica* Borkh.)^45^, sweet cherry (*Prunus avium* L.)^46^, and purple kale (*Brassica oleracea* var. *acephala* f. tricolor)^27^, low-temperature treatment during the growth process can induce the synthesis of anthocyanins, thereby increasing their nutritional quality, ornamental appearance, and cold tolerance. A highly inbred line, ‘Xiao Baojian’ (XBJ), was used to study the anthocyanin regulation pathway and its related genes under low-temperature stress. Consistent with previous studies, low temperature caused the leaves of Chinese cabbage to gradually turn purple^42,47^ (Fig. 1). At the beginning of low-temperature treatment, the anthocyanin content changed, although the phenotype had not yet appeared, indicating that anthocyanin regulation by low temperature is a gradual accumulation process in Chinese cabbage.

Phenotype-based transcriptome regulation research is an effective research method^48,49^. Transcriptome analysis refers to the analysis of the collection of all transcripts in a cell under a certain physiological condition. Using this analytical method, we previously studied the DEGs and related regulation pathways of anthocyanin synthesis in red leaf lettuce (*Lactuca sativa* L.)^50^ and mango (*Mangifera indica* L.)^51^. Given the unclear regulatory network of anthocyanins under cold stress, RNA-seq was performed on Chinese cabbage XBJ for four low-temperature treatment periods and five control periods. The comparison of DEGs in different periods of low-temperature stress and the comparison of DEGs in the same period showed that many cold-regulating genes are early response genes, and there were also many cold-regulating genes that were gradually regulated as low temperatures persisted (Fig. 3). Furthermore, 587 DEGs were enriched in the "biosynthesis of other secondary metabolites" pathway, and the "phenylpropanoid biosynthesis" (ko00940) contained 293 DEGs. "Flavonoid biosynthesis" (ko00941) and "anthocyanin biosynthesis" (ko00942) pathways were contained in the "biosynthesis of other secondary metabolites" pathway (Fig. 4), indicating that the anthocyanin biosynthesis pathway is active under low-temperature treatment.

Gene temporal expression patterns showed the enrichment pathways in Chinese cabbage under persistent low-temperature conditions (Fig. 5). DEGs of "regulation of autophagy," "SNARE interactions in vesicular transport," "endocytosis," "nicotinate and nicotinamide metabolism," "caffeine metabolism," and "non-homologous end-joining" KEGG pathways were gradually increased during persistent low-temperature conditions (Fig. 6a, b), indicating that DEGs of these pathways had resistance to cold stress and reduced the damage to Chinese cabbage caused by low temperatures. In contrast, the DEGs of "porphyrin and chlorophyll metabolism," "2-oxocarboxylic acid metabolism," "C5-branched dibasic acid metabolism," "ribosome," and "glucosinolate biosynthesis" had continuously lower expression as low-temperature conditions persisted (Fig. 6c, d), indicating that DEGs of these pathways are susceptible to low-temperature stress. These candidate pathways can be used for further research on Chinese cabbage under low-temperature stress.

Anthocyanins require three pathways for synthesis: phenylpropanoid (ko00940), flavonoid (ko00941), and anthocyanin biosynthesis (ko00942) pathways. Phenylpropanoid, flavonoid, and anthocyanin biosynthesis pathways were enriched in cluster 1, and flavonoid biosynthesis pathways were enriched in cluster 3 of gene temporal expression patterns (Fig. 7). The top 20 hub genes with the highest correlation relationships among clusters 1 (Fig. 7b) and 3 (Fig. 7d) were identified to further analyze the candidate genes of anthocyanins and their interaction genes under low-temperature conditions. Interestingly, five hub genes related to flavonoid biosynthesis (ko00941) were found in the top 20 genes of cluster 1: BraA09g042420.3C.gene (*F3H*), BraA09g019440.3C.gene (*DFR*), BraA01g013470.3C.gene (*ANS*), BraA10g030950.3C.gene (*FLS1*), and BraA03g050560.3C.gene (*ANS*) (Supplementary Table S7 and S9). F3H is a key enzyme in the biosynthetic pathway of plant anthocyanins; it can catalyze flavanones to produce dihydroflavonols. The *F3H* gene has been cloned in plants, such as *Arabidopsis thaliana*^52^, *Oryza sativa*^53^, and *Solanum tuberosum*^54^. Studies have shown that RNAi-mediated silencing of the *F3H* in strawberries, which blocks the biosynthesis of anthocyanins, in turn causing an uneven color distribution^55^. DFR is also a pivotal enzyme in flavonoid and anthocyanin biosynthesis, and under the action of the cofactor NADPH, the fourth carbon group is reduced to a hydroxyl group, which in turn catalyzes the synthesis of leuco anthocyanins from dihydroflavonols^56^. In many studies, the synthesis of anthocyanins is inseparable from the catalysis of DFR enzymes during the ripening process of flowers and fruit^57–60^. ANS is located at the end of the pathway of anthocyanidin synthesis and serves to catalyze the conversion of leucoanthocyanidin into colored anthocyanidin^61^. The *ANS* gene and its promoter sequence were cloned and obtained from *Forsythia suspensa* and demonstrated that the lack of anthocyanin accumulation in its petals is due to the lack of *ANS* gene expression^62^. FLS is a key enzyme for the synthesis of flavonols and is another branch of the flavonoid biosynthesis pathway^63^. *F3H*, *DFR*, and *ANS* were the most closely related genes involved in anthocyanin synthesis in Chinese cabbage under low-temperature conditions. Their expression level was much higher than the control (Fig. 9), indicating that *F3H*, *DFR*, and *ANS* play a vital role in resistance to low-temperature stress in Chinese cabbage.

Furthermore, the interaction networks of *DFR*, *ANS*, and *F3H* were screened and showed that flavonoid biosynthesis pathway genes (*DFR*, *ANS*, *F3H*, *FLS1*, *CHS1*, *CHS3*, and *TT8*) and defense mechanism-related genes (*DFR*, *SNL6*, and *TKPR1*) interact with each other (Fig. 8, Table 1). CHS is the first key enzyme for the synthesis of flavonoids in plants, which catalyzes the synthesis of p-coumaroyl-CoA to naringenin chalcone, which is the precursor of all flavonoid classes^64^. Previous studies have shown that the expression of *CHS* can affect flavonoid production. The inactivating mutation of *CHS* leads to a lower accumulation of flavonoids in *Arabidopsis*^65^. *CHS* overexpression can accelerate the synthesis of phenolics and anthocyanins in *Silybum marianum*^66^. Studies have shown that *BrTT8* has been recognized as a regulator of anthocyanin biosynthetic pathway genes in turnip (*Brassica rapa*; Brassicaceae)^67^, and inducing the bHLH regulator *BrTT8* at low temperature can activate late anthocyanin biosynthesis in purple Chinese cabbage^42^. Using a forward genetic screen, it was found that the SA signal pathway factor encoding the cinnamoyl-CoA reductase-like gene *SNL6* was necessary for rice pathogen-related protein expression and OsNPR1-dependent bacterial blight resistance^68^. The *TKPR1* in *Arabidopsis* was first thought to be involved in the flavonoid pathway^69^. Later studies found that *TKPR1* was very important for the fertility of male plants; the ostkpr1 mutant did not produce mature pollen or seeds in rice^70^. Interestingly, previous research indicated that suppressing *IbDFR* in sweet potato led to a decrease in anthocyanin accumulation and reduced tolerance to abiotic stress^71^. In our research, *DFR*, *SNL6*, and *TKPR1* were related to cell wall, membrane, and envelope biogenesis (Supplementary Table S9). Overall, flavonoid biosynthesis pathway genes (*DFR*, *ANS*, *F3H*, *FLS1*, *CHS1*, *CHS3*, and *TT8*) and defense mechanisms-related genes (*DFR*, *SNL6*, and *TKPR1*) interact with each other in low-temperature conditions. Low temperature caused the synthesis and accumulation of anthocyanins in Chinese cabbage and additionally activated defense mechanisms. Therefore, the synthesis of anthocyanins was also accompanied by the activation of defense mechanisms.

The expression levels of anthocyanin synthesis structural genes were compared in our experiment, and a heatmap of the expression levels of synthesis pathway genes was illustrated. (Fig. 9). Low temperature initially stimulated the phenylpropanoid biosynthesis pathway, modifying the expression of *PALs*, C4Hs, and 4CLs and thereby affecting the production of cinnamic acid, p-coumaric acid, and p-coumaroyl-CoA. PAL is a rate-limiting enzyme in the metabolic pathway of polyphenols and exists in almost all plant cells^72^. Studies have also shown that PAL is involved in the defense response of plant cells and resistance to biotic and abiotic stress^73,74^. C4H is the second key enzyme of the phenylpropanoid pathway, catalyzing cinnamic acid to produce p-coumaric acid. Studies have shown that *AaC4H* knockdown in *Artemisia annual* reduced the content of p-coumaric acid, total phenolics, and anthocyanins^75^. *4CL* converted p-coumaric acid to p-coumaroyl-CoA and then connected to the second step of anthocyanin synthesis—flavonoid biosynthesis. Low temperatures would stimulate EBGs (*CHS*, *CHI*, *F3H*, and *F3’H*), which would produce naringenin chalcone, naringenin, and dihydroflavonols. Finally, in the third step, the anthocyanin biosynthesis pathway involves LBGs (*DFR*, *ANS*, and *UGT75C1*). UGT75C1 is a late gene of anthocyanin synthesis, determining the glycosylation of anthocyanin in the process of biosynthesis, which determines the change in pigment intensity and color^11^. Here, low temperature affected the three pathways necessary for anthocyanin synthesis, and the expression of DEGs that promote anthocyanin synthesis increased during low temperature persistence. Moreover, defense mechanism genes (*SNL6* and *TKPR1*) and anthocyanin synthesis genes may have an interactive relationship, indicating that the effect of low temperature on the synthesis of Chinese cabbage anthocyanins is also a form of resistance.

The anthocyanin synthesis pathway is also regulated by many transcription factors, including those of the MYB gene family. In *Arabidopsis*, *AtMYB12* and *AtMYB111* in the flavonoid biosynthesis pathway have a similar structure and functionality to the EBGs of *AtCHS*, *AtCHI*, and *AtF3H*^76^. *AtMYB75*, *AtMYB113*, and *AtMYB114* mainly participate in the anthocyanin biosynthesis pathway to regulate the expression of LBGs (*F3’H*, *DFR*, *ANS*, and *UGT75C1*), and overexpression of *AtMYB75*, *AtMYB113*, and *AtMYB114* increased the anthocyanin content^77^. In this study, the expression levels of *MYB12*, *MYB75*, *MYB111*, *MYB113*, and *MYB114* were consistent with changes in the regulated structural genes of Chinese cabbage under low temperatures (Fig. 10). Hence, these MYBs might be associated with anthocyanin biosynthesis in Chinese cabbage under low-temperature conditions and be worthy of in-depth study. We randomly selected 14 structural genes in the anthocyanin synthesis pathway for consistency verification by qRT-PCR and RNA-seq (Fig. 11). We also selected two genes (BraA10g027720.3C.gene *FLC1*; BraA03g004170.3C.gene *FLC3*; downward trend)^39^ at low temperature to further verify the accuracy of the qRT-PCR work, and found that RNA-seq and qRT-PCR results were consistent during the low temperature process, indicating the reliability of RNA-seq. We found that most genes were most expressed in L-15DAT, and the purple phenotype was found at the beginning of L-15DAT. The expression of genes may have reached a certain level, activating the accumulation of anthocyanins, which slowly accumulated in the later stage.

## Supplementary Information

Supplementary Information 1.

Supplementary Information 2.

## Data Availability

The Illumina raw data were submitted to the Sequence Read Archive (SRA) database at NCBI under BioProject number PRJNA763246 under the following link: https://www.ncbi.nlm.nih.gov/bioproject/PRJNA763246.
