# Supplementary material for: Transcriptome analysis reveals anthocyanin regulation in Chinese cabbage (Brassica rapa L.) at low temperatures
Source: Sci Rep. 2022 Apr 15;12:6308. doi: 10.1038/s41598-022-10106-1 (PMC9012755; doi:10.1038/s41598-022-10106-1)
Supplement: Supplementary file 1 — Supplementary Information 1. [file 41598_2022_10106_MOESM1_ESM.docx]

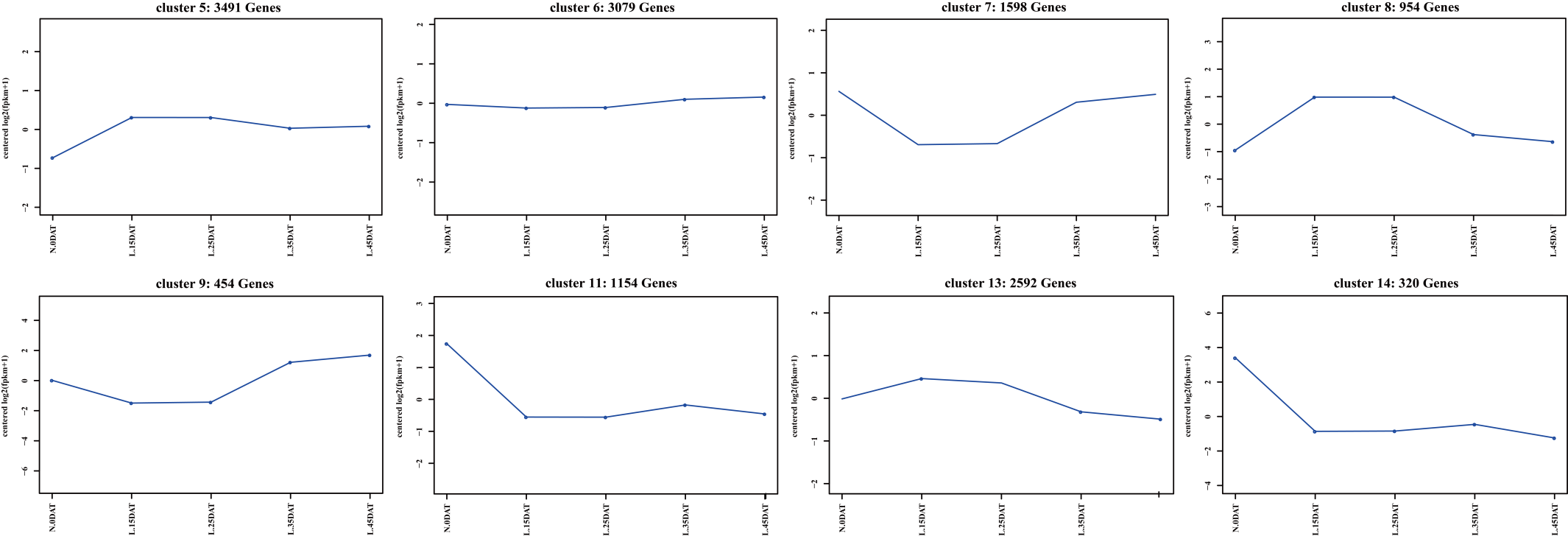


**Supplementary Fig. S1.** The other expression clusters of DEGs in low-temperature conditions. Each square represents one cluster. The x-axis represents treatment time, and the y-axis represents expression.
